# Evaluating undesired scratching in domestic cats: a multifactorial approach to understand risk factors

**DOI:** 10.3389/fvets.2024.1403068

**Published:** 2024-07-03

**Authors:** Yasemin Salgirli Demirbas, Joana Soares Pereira, Xavier De Jaeger, Laurianne Meppiel, Sarah Endersby, Gonçalo da Graça Pereira

**Affiliations:** ^1^Department of Physiology, Faculty of Veterinary Medicine, Ankara University, Ankara, Türkiye; ^2^Department of Psychology, University of Prince Edward Island, Charlottetown, PE, Canada; ^3^Egas Moniz Center for Interdisciplinary Research (CiiEM), Egas Moniz School of Health and Science, Almada, Portugal; ^4^Ceva Santé Animale, Libourne, France

**Keywords:** domestic cat, undesired scratching, cat behavior, environmental factors, behavioural characteristics

## Abstract

**Introduction:**

Despite being a natural feline behavior, scratching can become undesirable from a human perspective when directed at household items. This complex behavior can stem from various motivations, ranging from individual cat characteristics to environmental factors. This study investigates the factors influencing the increased level of undesirable scratching behavior in domestic cats, considering both cat-related and environmental aspects.

**Methods:**

Data from 1,211 cats were collected for this study. An online questionnaire comprising three sections was utilized. The first section gathered caregiver demographics, while the subsequent section examined aspects of cats’ daily routines, social interactions, environments, behaviours, and temperaments. The final section assessed the frequency and intensity of undesirable scratching behavior in cats. Scratching behavior was evaluated based on a combined scratching index.

**Results:**

The study suggests that the presence of a child may be associated with scratching episodes in the home environment. Additionally, factors such as play duration, playfulness, and nocturnal activity were identified as significant contributors to heightened scratching levels (*p* ≤ 0.05). Aggressiveness and disruptiveness also played significant roles in increased scratching behavior (*p* ≤ 0.05). The location of scratching posts emerged as a significant factor, with posts placed in areas frequented by the cat being more effective in redirecting scratching behavior (*p* ≤ 0.05).

**Discussion:**

This study reveals several significant associations between cat characteristics, nocturnal activity and play, as well as the environment. It underscores the multifaceted nature of undesirable scratching behavior and emphasizes the importance of comprehensively understanding both the individual characteristics of the cat and its environment to effectively address this behavior.

## 1 Introduction

The domestic cat exhibits sociability as a trait, forming and maintaining social bonds with humans despite not being socially dependent (1–3). As a result of common social history with humans for almost 10,000 years, they can easily adapt to human environments even though most of their behavioural biology still mimics that of their solitary ancestors (4). Domestic cats have a complex nature, as they are both social animals and possess strong territorial instincts. This complexity often leads people to misinterpret cats’ behavioural and environmental needs, regardless of their level of theoretical knowledge (5, 6). Scratching behavior, which is included in the normal behavioural repertoire of cats, is one of the most apparent examples of this misinterpretation, as this behavior is often perceived as a behavioural problem by caregivers (7, 8).

Undesired scratching, a behavior characterized by the destructive targeting of household items (9, 10), poses a significant threat to feline welfare, often prompting misperceptions and interventions within domestic settings. Caregivers often feel frustrated due to the destructive impact of scratching behavior on their living space (11). This frustration can heighten when both the cat’s stress levels and scratching behavior increase simultaneously in response to confrontational interventions such as positive punishment. In such cases, scratching behavior may serve as a marking behavior in reaction to heightened social tension (10, 12). This frustration may escalate to extreme measures taken by caregivers, such as onychectomy, relinquishment of the cat, or even euthanasia (8, 13). Despite widespread opposition from veterinary authorities to onychectomy (14), it remains a contentious approach for managing undesirable scratching, particularly when the alternatives involve euthanizing the cat (15, 16).

Several studies have investigated the main causes of undesired scratching by considering various aspects. Some studies examined environmental stressors (17) and needs of cats (10), while others focused on management strategies and demographics of cats and their caregivers (12, 18). The only proposed link between cat demographics and undesired scratching in these studies was age, i.e., a higher possibility of scratching was observed in younger cats (10, 12). The absence of suitable scratching materials in the environment and a lack of understanding of cat ethology are proposed as major contributors to the development of this problem (12). Positive punishment was further reported to be associated with increased level of scratching (12). On the other hand, the use of positive reinforcement methods (10) or feline synthetic pheromones (18–20) are suggested to help reduce undesired scratching.

In cat ethology, scratching serves many purposes such as maintaining claw health, provision of safety by marking and social communication (10, 21). Therefore, the underlying motivation of scratching is multifactorial and depends not only on social and physical factors but also on the individual cat. Given that scratching is an essential element of the natural behavior of cats, it is crucial to understand cat related factors that may cause an increase in intensity or frequency of this behavior, to ensure good welfare of cats.

This study aims to comprehensively evaluate undesired scratching by assessing the intensity and frequency of this behavior, as opposed to assessing its presence vs. absence. Furthermore, the study seeks to shed light on the risk factors related to cat behaviours and environmental factors that may be associated with increased scratching levels in the home environment. The hypothesis posits that certain behavioural characteristics of cats could be significantly linked to higher levels of scratching behavior. Thus, this research seeks to offer a deeper understanding of the reasons behind undesired scratching, ultimately providing valuable insights for the development of more effective and tailored interventions to enhance the well-being of cats and strengthen their bonds with their human companions.

## 2 Materials and methods

### 2.1 Study design

This study reports the further analysis of data from a blinded, randomized and controlled consumer study which has already been published (18). The protocol underwent review by the ethics committee, and the collection of personal data adhered to all relevant legal obligations concerning the protection of personal data and privacy. This included compliance with the Law of 1978 and the GDPR, as well as following the recommendations and guidelines of the Commission Nationale Informatique et Libertés (CNIL).

### 2.2 Questionnaire design

To collect relevant data, a questionnaire which encompassed a total of three main parts was designed. Part I focused on caregiver demographics and Part II explored diverse facets of the daily lives and behavior of cats. This included their daily schedules, social engagements, physical surroundings, behaviours and temperament. Part III centered on the level of the undesired scratching behavior displayed by the cats. This part included questions regarding the frequency and intensity of undesired scratching events. To assess the frequency of undesired scratching, caregivers were instructed to recall any instances they directly witnessed or inferred from new damage noticed over the past week. A semi-quantitative scale ranging from 0 (Never) to 6 (Multiple times per day) was used to measure the frequency of scratching. Additionally, caregivers rated the intensity of scratching on a visual analog scale (VAS) from 1 to 10, considering factors like duration and damage extent. A minimum intensity rating of 1 was mandated since any scratching activity (excluding never) was presumed to possess some degree of intensity. This methodology mitigated bias in the Global Index Score, ensuring that any frequency greater than zero corresponded to a certain level of intensity (Please see the Appendix for the questionnaire).

### 2.3 Participants

The study was conducted in France, and the participants were recruited from a panel of cat caregivers. Potential participants were contacted via email and had to meet specific inclusion criteria to participate in the study. The inclusion criteria were as follows: (1) being 18 years or older and providing signed consent to participate, (2) owning a cat that presented undesired scratching for at least 1 month, with a minimum frequency of two episodes per week (indoor scratching), (3) having only one cat to avoid potential stress from multi-cat households, and (4) having at least one scratching post, cat tree, or another scratching device that was consistently available to the cat in order to reliably evaluate the extent of scratching on household items.

### 2.4 Questionnaire distribution

The questionnaire was distributed via a web application to caregivers, who were notified of their availability by email. The participants received the eligibility questionnaire by email with the selection questions and were asked if they would like to participate in the study. Reminders were sent via email and SMS 24 h after questionnaire availability, giving caregivers another 24 h to complete it.

### 2.5 Ethical considerations

The study was conducted following ethical guidelines, and informed consent was obtained from all participants. The anonymity of respondents was maintained throughout the questionnaire process to ensure confidentiality and encourage honest responses.

### 2.6 Statistical analysis

SAS 9.4 was utilized as a statistical software program. The collected questionnaire data were analyzed to explore the associations between various factors and undesired scratching behavior in domestic cats.

The scratching behavior levels for frequency, intensity, and index of each cat were categorized. In the assessment of frequency, a scale between 0 and 6 was used, and cat caregivers were asked to mark the appropriate option regarding the frequency of scratching behavior of their cats (6: Every day, more than twice a day; 5: Every day, once or twice a day; 4: Almost every day; 3: Every other day; 2: Twice a week; 1: Once a week; 0: Never). Intensity was assessed using a Visual Analog Scale (VAS), which is a subjective measurement tool. On this scale, participants were asked to rate the intensity of scratching. The scale ranges from 1 to 10, where 1 represents an extremely low intensity, and 10 represents an extremely high intensity of scratching. The scratching index, which is obtained by multiplying the frequency and intensity is used to combine two different aspects of the scratching behavior to create a single value (22). The Cat Behavior Issues Assessment Scale (CABIAS™) is a validated scale used to assess common problem behaviours in cats, such as urine marking, scratching, fear, and issues related to cohabitation between cats (23). CABIAS employs an index score as a scoring system that combines aspects of the frequency and intensity of the problem behavior based on the previous week of observation.

Descriptive analyses were conducted, and comparisons of risk factors and cat characteristics based on scratching behavior levels (low and high) were performed using statistical tests. All qualitative parameters were assessed with a Chi-square test, while the sole quantitative parameter (weight) was analyzed using a Wilcoxon test. Qualitative parameters encompassed questions pertaining to cat characteristics including sociability, disruptiveness, lethargy, vivacity, boisterousness, and tranquility, alongside their daily routines such as play, activity levels, grooming habits, and dietary patterns. Additionally, aspects related to behavior, such as biting history, litter box issues, and preferred scratching locations, were also considered within the qualitative framework. Environmental elements, such as the placement of scratching posts and the presence of children, as well as demographic information including breed, gender, neutering status, and Body Condition Score (BCS), were further explored as qualitative parameters (For detailed questionnaire information, please refer to the Appendix).

## 3 Results

### 3.1 Study population

This study was a sub-study of a previously published controlled customer study (18). In this study, exclusive focus was placed on the data collected on Day 0, serving as the initial baseline before any treatment was administered to the cats. The study population consisted of 1,211 cats. Out of the initially recruited 1,415 cats, 204 were excluded due to factors such as changes in residence, missing baseline data, or unavailability of a scratching post.

Cats were divided into three groups, each with a similar number of individuals, based on the distribution of a specified index. The intermediate group (*N* = 356) was excluded from further analysis, resulting in the creation of two distinct categories representing cats with either high or low scratching behavior. This separation was done to enable a more straightforward comparison between cats exhibiting low and high levels of scratching. Such categorization aligns with the study’s goal of examining factors associated with extreme scratching behavior, concentrating the research question on these specific behavioural categories (Table 1).

**Table 1:** Index distribution.

Parameter	Statistics	Total (*N* = 1,211)
Index – scratching level		
Low<=20	*n* (%)	361 (29.8%)
High> = 30	*n* (%)	494 (40.8%)

### 3.2 Cat demographics and characteristics

No significant difference was observed between purebred and mixed-breed cats, gender, neutering status, body condition score, and actual weight concerning the scratching index (*p* ≥ 0.05).

**Figure 1:**
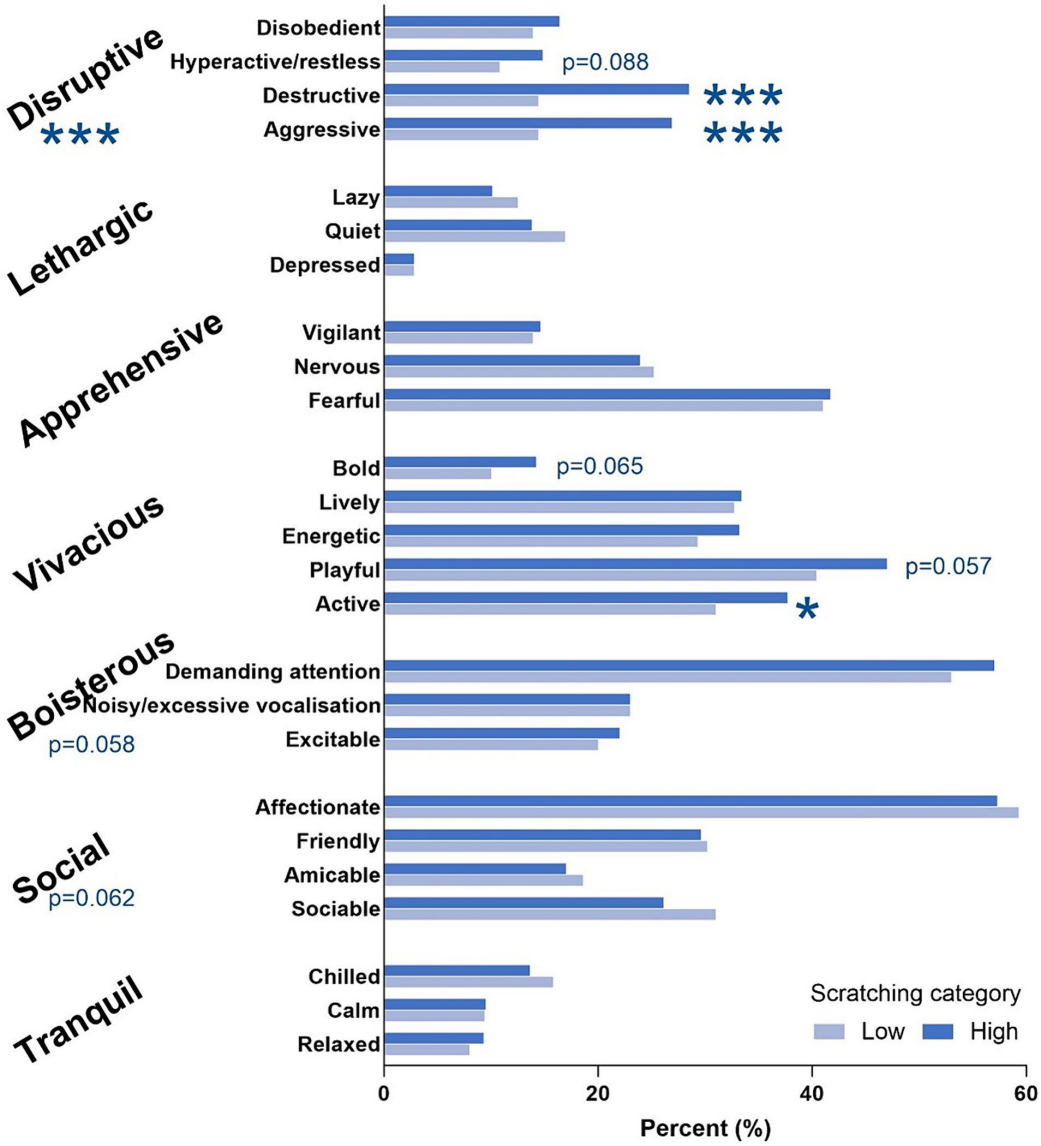
Cat characteristics according to level of index scratching.

All questions related to the characteristics of the cats were categorized into seven main groups: disruptive, lethargic, apprehensive, vivacious, boisterous, social, and tranquil (Figure 1). Disruptiveness was found to be significantly related to a high scratching level (*p* ≤ 0.01). Moreover, sub-characteristics such as aggressiveness and destructiveness were also found to be significantly associated with a high scratching level (*p* ≤ 0.01). No other main or sub-characteristic had a significant effect on scratching level except for the sub-characteristics active (*p* ≤ 0.05). The main characteristic ‘boisterous’ and the sub-characteristic “playful” showed a trend towards significance (*p* = 0.058). In the high scratching cat profile group, 58% of the cats were characterized as disruptive. In the high scratching cat profile group, 58% of the cats were characterized as disruptive.

### 3.3 Daily routines and environment

**Figure 2:**
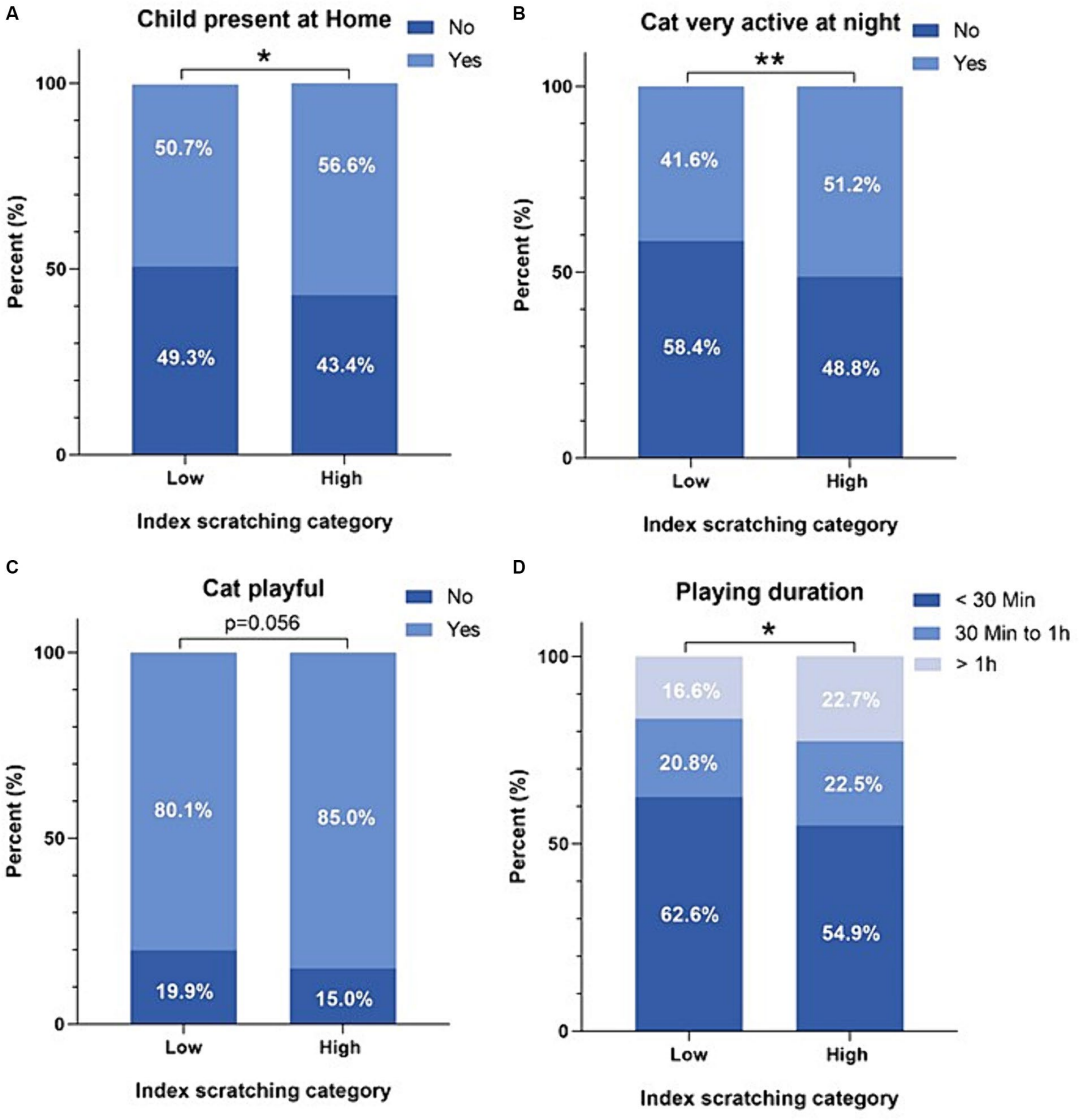
Effect of different factors (**A**: Child presence at home, **B**: Cat activity level at night, **C**: Playfulness of the cat, **D**: Playing duration) on low and high scratching profiles.

The analysis of our study revealed significant effects of different factors on scratching behavior among the subjects. The duration of play, nocturnal activity, the presence of children (*p* ≤ 0.05; Figure 2), as well as the placement of the cat tree, the availability of scratching posts, and preferences for scratching areas were identified as factors significantly associated with the level of scratching (*p* ≤ 0.05; Table 2).

**Table 2:** Answers related to scratching location.

Question	Possible answer	Scratcher category (%)		*p* value
		Low	High	
Regarding the location of the scratching post it is in:	The same room as the behavior problem happens	65.7%	74.7%	0.00404
	A different room from where the behavior problem happens	34.3%	25.3%	
Does your cat use the cat tree / scratching post / other scratching device that you provide to him?	No	25.2%	37.0%	0.00025
	Yes	74.8%	63.0%	
Is the cat tree / scratching post / other scratching device always available for your cat to use?	No	4.7%	2.2%	0.04397
	Yes	95.3%	97.8%	
Where does your cat scratch most?	In many different places	32.1%	57.9%	<0.0001
	Only on curtains	1.1%	0.2%	
	Only on sofas	32.4%	26.7%	
	Only on chairs	7.5%	3.0%	
	Only on carpets/mats/rugs	7.2%	4.0%	
	Only on furniture	5.5%	1.6%	
	Other	14.1%	6.5%	

## 4 Discussion

The present study aimed to investigate the factors influencing the level of undesired scratching behavior in domestic cats, adopting a multifactorial approach that considered various behavioural and environmental aspects. The findings reveal a significant association between specific factors and cats in high scratching profiles.

One noteworthy finding is the influence of the presence of a child at home on the high level of scratching behavior. It appears that the presence of a child in the household could potentially contribute to heightened stress levels, thereby leading to more frequent and intense scratching episodes (24). This outcome aligns with previous research suggesting that the presence of children, particularly during specific developmental stages, might amplify the likelihood of undesirable scratching incidents within the home (25). Conversely, it is crucial to highlight that presence of children have been documented as a prominent factor behind the relinquishment or return of adopted pets (26, 27). While most studies predominantly concentrate on the well-being and health of the human residents sharing the same household with cats, these findings underscore the significance of evaluating the quality of life for both constituents – humans and pets – to ensure the establishment of a harmonious environment. One additional factor that should be discussed here is that, within the scope of this study, no detailed investigation has been conducted regarding children-cat interaction. Specifically, the presence of either the child or the cat at the beginning, as well as the age of the children interacting with the cat, was not examined. Consequently, further research is required to determine whether the cat’s behavior is influenced by the arrival of a new child as a family member or is generally associated with the intrinsic presence of the child. Additionally, exploring whether the age of the child contributes to scratching behavior is a factor that warrants further investigation.

Similar to a recent study (28), this study also underscores the importance of play behavior in preventing undesired problem behaviours exhibited by cats in the household environment. According to the findings of this study, factors such as the duration of play, playfulness, and nocturnal activity were identified as influencing the level of scratching behavior. This suggests that cats characterized by increased playfulness and engaged in prolonged play and activity sessions tend to exhibit heightened scratching activities. A potential explanation for the association between heightened activity/play and increased scratching involves sustained sympathetic arousal, which may be connected to vigilance and stress, thereby amplifying marking-induced scratching behavior (29, 30). Play itself holds a crucial role in the well-being of cats, serving as an outlet for their inherent hunting and exploration instincts (31, 32). Nevertheless, in the wild, individual play is intertwined with predation, necessitating a heightened arousal level for repetitive yet brief periods (33, 34). The observed association between intensified play and heightened nocturnal activity may suggest prolonged or inappropriate play routines for cats, potentially leading to increased stress and irritability. This result may underscore the significance of implementing appropriate play routines and periods for cats as it is stated above (32).

Inadequate or insufficient play and hunting opportunities may further result in frustration, prompting increased scratching as a stress release mechanism (35, 36). Additionally, frustration can serve as a potential underlying factor for disruptive behavior, which is in turn associated with a higher level of scratching behavior. This observation posits that cats with a lower threshold for frustration may exhibit heightened scratching responses as a manifestation of stress, or the environment’s failure to meet basic needs may induce frustration, leading to elevated aggressiveness and disruptiveness (37). Emphasizing the significance of the five pillars of cat care, including the provision of appropriate play opportunities (34), is crucial. Promoting regular and brief interactive play sessions, coupled with offering suitable toys, can alleviate stress and consequently reduce undesirable scratching behaviours.

In this study, the location of the scratching post emerged as a significant factor influencing scratching behavior, It was further revealed that the scratching post was situated in the same room where scratching occured for both low and high level scratchers. Given that scratching behavior in cats typically manifests in socially significant areas, it may be inferred that the motivation behind this behavior serves as a means of expressing their underlying emotional state (38, 39). Cats may prefer specific locations for scratching that align with their territorial and marking patterns. Providing well-positioned scratching posts in areas frequented by the cat may help redirect scratching to more appropriate surfaces, reducing damage to household items (40).

While this study provides valuable insights into scratching behavior in domestic cats, it is important to acknowledge several limitations. Firstly, relying on caregiver-reported data introduces potential biases due to subjective interpretation and recall biases inherent in such reports. However, it is worth noting that the study assessed scratching behavior based on observations over the previous 7 days, which may help mitigate bias stemming from caregivers’ memory. Additionally, previous research has demonstrated the reliability of the assessment scale across different caregivers within households, suggesting that memory-related biases may not significantly impact the scores (23). Moreover, given the significant role of caregiver perspectives in shaping feline welfare, bias resulting from caregiver perception may still offer valuable insights into factors related to undesired scratching. Even if this assessment relies on caregiver reports, it is important to keep in mind that this behavior is never directly assessed by the veterinarian and is always based on caregiver reports or complaints. Another limitation lies in the adoption of a cross-sectional research design. Although this design allows for identifying associations between factors and scratching behavior, longitudinal investigations are crucial for understanding the temporal dynamics and causal pathways underlying undesired scratching behavior in domestic cats. Lastly, the lack of detailed exploration into the nuances of children-cat interaction, including factors such as the age of the child and the timing of their introduction to the cat, limits our understanding of their influence on scratching behavior in domestic cats. Addressing these limitations in future studies is essential for advancing our comprehensive understanding of the multifaceted factors contributing to undesired scratching behavior in the home environment.

In conclusion, this study unveils the intricate and multifaceted nature of undesired scratching behavior in domestic indoor cats. Gaining insights into these contributing factors is paramount for cat caregivers, as it enables the implementation of targeted interventions to encourage appropriate scratching and improve the overall well-being of their feline companions. The pivotal role of addressing both the physical and social needs of cats emerges as a critical strategy in mitigating undesirable behaviours. This holistic approach ensures a comprehensive understanding and effective management of scratching-related issues in domestic cats.

## Data availability statement

The raw data supporting the conclusions of this article will be made available by the authors, without undue reservation.

## Ethics statement

The animal studies were approved by Ceva Santé Animale Committee (ref CFAEC-2022-08). The studies were conducted in accordance with the local legislation and institutional requirements. Written informed consent was obtained from the owners for the participation of their animals in this study.

## Author contributions

YD: Conceptualization, Investigation, Methodology, Supervision, Writing – original draft, Writing – review & editing. JP: Conceptualization, Methodology, Supervision, Writing – review & editing. XJ: Data curation, Formal analysis, Methodology, Visualization, Writing – review & editing. LM: Data curation, Formal analysis, Software, Visualization, Writing – review & editing. SE: Conceptualization, Methodology, Writing – review & editing. GG: Conceptualization, Data curation, Investigation, Methodology, Supervision, Writing – review & editing.
