# Supplementary material for: Evaluating undesired scratching in domestic cats: a multifactorial approach to understand risk factors
Source: Front Vet Sci. 2024 Jul 3;11:1403068. doi: 10.3389/fvets.2024.1403068 (PMC11251885; doi:10.3389/fvets.2024.1403068)
Supplement: Supplementary file 1 [file Data_Sheet_1.PDF]

Appendix 3: Questionnaire

1- Recruitment questionnaire

We require you to agree to respect a confidentiality agreement relating to this study. This agreement states that you shall keep the information that you see confidential. This includes posting details of the study on the internet (Facebook, twitter, social media, forums, chats, blogs, etc...) or openly discussing the details of the study with anyone other than a representative of this company. We take this agreement seriously and ask you to do so as well.

Please consider the problematic scratching and the term “scratching” only when this behaviour happens outside of the places that you designated for your cat to scratch on (e.g. if this happens on courtins, sofas, chairs, beds, seats, carpets and other furniture). Scratching on cat trees / scratching posts /other scratching devices is not considered as a problem and thus must not be considered in your answers unless we ask about it. Scratching people is also not considered.

1. Do you agree or disagree to respect this confidentiality agreement?
    1. I agree
    2. I don’t agree

STOP if 2 quoted

2. Does any of the following apply to you? (SELECT ALL THAT APPLY)
    1. You belong to a research panel dedicated to a specific brand or a specific product manufacturer
    2. You are a trained panelist for sensory descriptive profiling of products
    3. None of the above

STOP if 1 or 2 quoted

3. Do you, or does any of your relatives, work in one of these areas? (SELECT ALL THAT APPLY)

Agribusiness	1	

1- Questionnaire de recrutement

Nous vous demandons de vous engager à respecter un accord de confidentialité relatif à cette étude. Cet accord stipule que vous devez garder confidentielles les informations que vous voyez. Cela inclut la publication des détails de l'étude sur Internet (Facebook, twitter, médias sociaux, forums, chats, blogs, etc...) ou la discussion ouverte des détails de l'étude avec toute personne autre qu'un représentant de cette société. Nous prenons cet accord au sérieux et vous demandons de faire de même.

Veuillez considérer les griffades problématiques et le terme "griffades" uniquement lorsque ce comportement se produit en dehors des endroits que vous avez désignés pour que votre chat se gratte (par exemple si cela se produit sur des courtines, canapés, chaises, lits, sièges, tapis et autres meubles). Le fait de se gratter sur les arbres à chat, les griffoirs ou autres dispositifs de griffades n'est pas considéré comme un problème et ne doit donc pas être pris en compte dans vos réponses, sauf si nous vous le demandons. Le fait de gratter les gens n'est pas non plus pris en compte.

1. Acceptez-vous ou non de respecter cet accord de confidentialité ?
3. Je suis d'accord
4. Je ne suis pas d'accord.

STOP si 2 cités

2. L'une des situations suivantes s'applique-t-elle à vous ? (SÉLECTIONNEZ TOUTES LES RÉPONSES QUI S'APPLIQUENT)
4. Vous faites partie d'un panel de recherche dédié à une marque ou à un fabricant de produits spécifiques.
5. Vous êtes un panéliste qualifié pour le profilage descriptif sensoriel des produits.
6. Aucune de ces réponses

STOP si 1 ou 2 cités

3. Est-ce que vous, ou l'un de vos proches, travaillez dans l'un de ces domaines ? (SÉLECTIONNEZ TOUTES LES RÉPONSES QUI S'APPLIQUENT)

Agribusiness	1	

Banking / Finance / Insurance	2	
Pharmaceutical industry	3	STOP
The cosmetic industry	4	
A veterinary practice / clinic	5	STOP
A pet store / pet shop	6	STOP
A survey / market research institute	7	STOP
An advertising / marketing / communication agency	8	STOP
Journalism / media	9	STOP
Automobile industry	10	
The manufacture or distribution of pet food or pet care products	11	STOP
None of these areas	12	

4. How old are you?

STOP if less than 18 yo

5. Does yourself or does someone else in your household suffer from a health problem or allergy?
    1. Yes
    2. No

STOP if 1 quoted

6. Do you have a cat at home?
    1. Yes
    2. No

STOP if 2 quoted

7. How many cats do you have at home? (SELECT ONE)
    1. 1
    2. 2
    3. 3 or more

Banque / Finance / Assurance	2	
Industrie pharmaceutique	3	STOP
L'industrie cosmétique	4	
Un cabinet ou une clinique vétérinaire	5	STOP
Une animalerie / un magasin pour animaux	6	STOP
Un institut de sondage / d'études de marché	7	STOP
Une agence de publicité / marketing / communication	8	STOP
Journalisme / médias	9	STOP
Industrie automobile	10	
la fabrication ou la distribution d'aliments pour animaux de compagnie ou de produits de soins pour animaux de compagnie	11	STOP
Aucun de ces domaines	12	

4. Quel âge avez-vous ?

STOP si moins de 18 ans

5. Est-ce que vous-même ou une autre personne de votre foyer souffre d'un problème de santé ou d'une allergie ?
    1. Oui
    2. Non

STOP si 1 cité

6. Vous avez un chat à la maison ?
    1. Oui
    2. Non

STOP si 2 cités

7. Combien de chats avez-vous à la maison ? (CHOISIR UN)
    1. 1
    2. 2
    3. 3 ou plus

STOP if code 2 or 3

8. Do you have other Pets at Home ?
   1. Dog
   2. Other
   3. No

9. Yourself, in general, excluding holidays, do you spend at least 4 hours awake (without sleeping) at home?
   1. No never
   2. Yes, 1 day by week
   3. Yes, 2 days by week
   4. Yes, 3 days by week
   5. Yes, 4 days by week
   6. Yes, 5 days by week
   7. Yes, 6 days by week
   8. Yes, everyday

Stop if code 1, 2, 3, 4 or 5

10. Are you the one in the household who cares of your cat most often (e.g. feeding, visit to the vet, administration of medication, etc.)?
   1. Yes
   2. No

11. How old is your cat? (SELECT ONE)
   1. 0-6 months (kitten)
   2. 7 months-2 years (junior)
   3. 3-6 years (adult)
   4. 7-10 years (mature adult)
   5. 11-14 years (senior)
   6. 15 years + (super senior)

STOP if 1, 5 or 6

STOP si code 2 ou 3

8. Avez-vous d'autres animaux domestiques à la maison ?
   1. Chien
   2. Autre
   3. Non

9. Vous-même, en général, hors vacances, passez-vous au moins 4 heures éveillé (sans dormir) à la maison ?
   1. Non, jamais.
   2. Oui, 1 jour par semaine
   3. Oui, 2 jours par semaine
   4. Oui, 3 jours par semaine
   5. Oui, 4 jours par semaine
   6. Oui, 5 jours par semaine
   7. Oui, 6 jours par semaine
   8. Oui, tous les jours

Arrêt si code 1, 2, 3, 4 ou 5

10. Êtes-vous la personne du foyer qui s'occupe le plus souvent de votre chat (par exemple, nourriture, visite chez le vétérinaire, administration de médicaments, etc.)
   1. Oui
   2. Non

11. Quel âge a votre chat ? (CHOISISSEZ UN)
   1. 0-6 mois (chaton)
   2. 7 mois-2 ans (junior)
   3. 3-6 ans (adulte)
   4. 7-10 ans (adulte mature)
   5. 11-14 ans (senior)
   6. 15 ans + (super senior)

STOP si 1 ou 6

12. Which ONE of the following best corresponds to the lifestyle of your cat? (SELECT ONE)<br>1. My cat lives exclusively indoors (with or without access to balcony)<br>2. My cat spends an average of 2 to 4 hours outdoors (terace/garden/street) where he can also scratch<br>3. My cat spends and average of 4 to 6 hours outdoors (terrace/garden/street) where he can also scratch<br>4. My cat spends in average more than 6 hours outdoors (terrace/garden/street) where he can also scratch<br>5. My cat lives exclusively outdoors (without access to the indoor)<br>STOP if 5 quoted	12. Lequel des éléments suivants correspond le mieux au mode de vie de votre chat ? (CHOISIR UN)<br>1. Mon chat vit exclusivement à l'intérieur (avec ou sans accès au balcon)<br>2. Mon chat passe en moyenne 2 à 4 heures à l'extérieur (terrain/jardin/rue) où il peut également faire ses griffes.<br>3. Mon chat passe en moyenne 4 à 6 heures à l'extérieur (terrasse/jardin/rue) où il peut également faire ses griffes.<br>4. Mon chat passe en moyenne plus de 6 heures à l'extérieur (terrasse/jardin/rue) où il peut également faire ses griffes.<br>5. Mon chat vit exclusivement à l'extérieur (sans accès à l'intérieur).<br>STOP si 5 cité
13. Does your cat or does one of your pets suffer from a health problem or allergy diagnosticated by a vet?<br>1. Yes<br>2. No<br>STOP if 1 quoted	13. Votre chat ou l'un de vos animaux domestiques souffre-t-il d'un problème de santé ou d'une allergie diagnostiquée par un vétérinaire ?<br>1. Oui<br>2. Non<br>STOP si 1 cité
14. Has your cat returned home after hospitalization, staying in a cattery or shelter in the previous 15 days?<br>1. Yes<br>2. No<br>STOP if 1 quoted	14. Votre chat est-il rentré à la maison après une hospitalisation, un séjour dans une chatterie ou un refuge au cours des 15 derniers jours ?<br>1. Oui<br>2. Non<br>STOP si 1 cité
15. Has your cat been diagnosed with arthritis or any other physical problem that affects his/her gait and/or causes physical discomfort?<br>1. Yes<br>2. No<br>STOP is 1 quoted	15. Votre chat a-t-il été diagnostiqué avec de l'arthrite ou tout autre problème physique qui affecte sa démarche et/ou lui cause un inconfort physique ?<br>1. Oui<br>2. Non<br>STOP est 1 cité
16. Is your cat recovering from orthopedic surgery (went under surgery less than 6 weeks ago) OR is his behaviour different (sick, prostrated…)<br>1. Yes<br>2. No<br>STOP if 1 quoted	16. Votre chat se remet-il d'une chirurgie orthopédique (il a été opéré il y a moins de 6 semaines) OU son comportement est-il différent (malade, prostré...) ?<br>1. Oui<br>2. Non<br>STOP si 1 cité

17. How many cat trees/scratching posts / other scratching device do you have at home? (SELECT ONE)

1. None
2. 1
3. 2 and more

STOP if code 1

18. Do you consider that your cat display undesirable scratching on vertical surfaces INDOORS other than the scratching post (such as sofa, curtains, furniture ...)

1. Yes
2. No

STOP if 2

19. In the last 7 days, how often have you noticed your cat has made inappropriate scratches (Scratching indoors on vertical surfaces other than the cat tree/scratching post/other scratching device you provide him e.g. sofa, furniture, curtains, door frames...)? The inappropriate scratching could be directly observed or deduced from new damage observed.

6. Every day, more than twice a day
5. Every day, once or twice a day
4. Almost every day
3. Every other day
2. twice a week
1. Once a week
0. Never

STOP if code 1or 0

20. Did this problem started to occur more than one month ago?

1. Yes
2. No

STOP if code 2

21. How long has this scratching been a problem?

17. Combien d'arbres à chat/de griffoirs/autres dispositifs pour griffer avez-vous à la maison ? (CHOISISSEZ-EN UN)

1. Aucun
2. 1
3. 2 et plus

STOP si code 1

18. Considérez-vous que votre chat fait preuve d'un comportement indésirable en griffant des surfaces verticales à l'INTÉRIEUR autres que le griffoir (canapé, rideaux, meubles, etc.) ?

1. Oui
2. Non

STOP si 2

19. Au cours des 7 derniers jours, combien de fois avez-vous remarqué que votre chat a fait des griffades inappropriées (griffades à l'intérieur sur des surfaces verticales autres que l'arbre à chat/le griffoir/autre dispositif de griffades que vous lui fournissez, par exemple canapé, meubles, rideaux, cadres de porte...) ? Les griffades inappropriées peuvent être observées directement ou déduits en raison de nouveaux dommages observés.

6. Tous les jours, plus de deux fois par jour
5. Tous les jours, une ou deux fois par jour
4. Presque tous les jours
3. Tous les deux jours
2. deux fois par semaine
1. Une fois par semaine
0. Jamais

STOP si code 1ou 0

20. Ce problème a-t-il commencé à se produire il y a plus d'un mois ?

3. Oui
4. Non

STOP si code 2

21. Depuis combien de temps ces griffades sont-elles un problème ?

Month/year drop-down menu<br><br>22. Have you consulted a veterinarian and/or a behaviourist because of this scratching problem ?<br>1. Yes<br>2. No<br><br>STOP if 1 quoted<br><br>23. Are you currently using OR have you been using the following products in the past 6 months, to help with the bad/annoying habits of your cat?<br>1. Yes, FELIWAY C*lassic* diffuser<br>2. Yes, FELIWAY C*lassic* spray<br>3. Yes, FELIWAY *MultiCat* diffuser<br>4. Yes, THUNDEREASE for cats<br>5. Yes, THUNDEREASE Multicat<br>6. Yes, COMFORT ZONE Calming diffuser<br>7. Yes, COMFORT ZONE Multi Cat diffuser<br>8. Yes, SENTRY Calming diffuser<br>9. Yes, nutraceuticals or medical calming products<br>10. Yes, other calming products, please specify<br>11. Do not know<br>12.No, none of those products<br>STOP if 12 not quoted and any of the others has been choosen<br><br>For this test you will be asked to test a pheromone diffuser intended to naturally soothe cats.<br><br>This product is aimed at naturally calming cats and helping to reduce signs of stress like scratching, peeing, hiding, etc.... This product is safe and veterinary recommended, has no sedative effect and no contra-indications.	Menu déroulant mois/année<br><br>22. Avez-vous consulté un vétérinaire et/ou un comportementaliste à cause de ce problème de griffades ?<br>1. Oui<br>2. Non<br><br>STOP si 1 cité<br><br>23. Utilisez-vous actuellement OU avez-vous utilisé les produits suivants au cours des 6 derniers mois, pour aider à corriger les mauvaises habitudes de votre chat ?<br>1. Oui, le diffuseur FELIWAY *Classic*<br>2. Oui, le spray FELIWAY *Classic*<br>3. Oui, le diffuseur FELIWAY *MultiCat*<br>4. Oui, THUNDEREASE pour les chats<br>5. Oui, THUNDEREASE Multicat<br>6. Oui, le diffuseur apaisant COMFORT ZONE<br>7. Oui, le diffuseur Multi Cat de COMFORT ZONE<br>8. Oui, le diffuseur apaisant SENTRY<br>9. Oui, produits nutraceutiques ou médicaux calmants<br>10. Oui, autres produits calmants, veuillez préciser<br>11. Je ne sais pas<br>12.non, aucun de ces produits<br>STOP si le 12 n'est pas cité et que l'un des autres a été choisi.<br><br>Pour ce test, il vous sera demandé de tester un diffuseur de phéromones destiné à apaiser naturellement les chats.<br><br>Ce produit a pour but de calmer naturellement les chats et d'aider à réduire les signes de stress comme les griffades, le marquage urinaire, les peurs excessives, les difficultés de cohabitation, etc. Ce produit est sûr et recommandé par les vétérinaires, il n'a aucun effet sédatif et aucune contre-indication.

24. Do you agree or disagree to test this product?

1. I agree
2. I don’t agree

STOP if 2 quoted

Further, by participating in this test, you undertake:

- To plug in this diffuser in the room where your cat spends most of his/her time (even of this is not the place where he/she scratches) for 28 days
- To complete 5 questionnaires: the day of the plugging, 7 days later, 14 days later, 21 days later and 28 days later
- You will only consider the problematic scratching when this behaviour happens outside of the places that you designated for your cat to scratch on (e.g. if this happens on courtins, sofas, chairs, beds, seats, carpets and other furniture).

This diffuser CANNOT be plugged:

- near any heater or electrical device.
- close to any window or air conditioning system.
- in a multi socket, multiplug adaptor or extension lead
- in a wall outlet with furniture or any object protruding from the wall above.

25. In which room does your cat spend most of his/her time?

1-In the kitchen
2-In the living room
3-In one bedroom
4 – in the hall
5 – outdoors
6 -Other, specify

STOP if 5 is quoted

26. What is the size of this room (in square meter)?

/ _____________ /

STOP if more than 70 M2

24. Êtes-vous d'accord ou non pour tester ce produit ?

1. Je suis d'accord
2. Je ne suis pas d'accord.

STOP si 2 cité

En outre, en participant à ce test, vous vous engagez :

- Brancher ce diffuseur dans la pièce où votre chat passe le plus de temps (même si ce n'est pas l'endroit où il fait ses griffes) pendant 28 jours.
- Remplir 5 questionnaires : le jour du branchement, 7 jours plus tard, 14 jours plus tard, 21 jours plus tard et 28 jours plus tard.
- Vous ne considérerez les griffades problématiques que lorsque ce comportement se produit en dehors des endroits que vous avez désignés pour que votre chat fasse ses griffes (par exemple, si cela se produit sur les courtines, les canapés, les chaises, les lits, les sièges, les tapis et autres meubles).

Ce diffuseur NE PEUT PAS être branché :

- près d'un appareil de chauffage ou d'un appareil électrique.
- à proximité d'une fenêtre ou d'un système de climatisation.
- dans une multiprise, un adaptateur multiprise ou un câble de rallonge
- dans une prise murale avec un meuble ou tout objet dépassant du mur au-dessus.

25. Dans quelle pièce votre chat passe-t-il le plus clair de son temps ?

1-Dans la cuisine
2-Dans la salle de séjour
3-Dans une chambre
4 - dans le hall
5 - à l'extérieur
6 -Autres, précisez

STOP si 5 est cité

26. Quelle est la taille de cette pièce (en mètres carrés) ?

/ _____________ /

STOP si plus de 70 M2

27. Do you have a free outlet in this room with no close heater or electrical device, no close window or air conditioning system, no close furniture or any object protruding from the wall above?

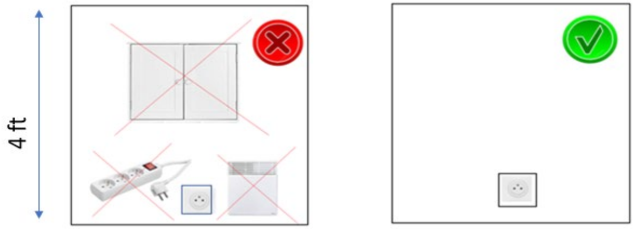

1-Yes
2-No
STOP if code 2

28. Do you intend to go on holiday or leave your home from the .. to… (duration of the test)?

1. No, don't intend to go away
2. Yes but not more than 2 days or just a week-end
3. Yes, at least 3 or 4 days

STOP if code 3

29. Are you?
1 – A woman
2 – A man

30. Do you live?
1. In an urban area
2. In the countryside

31. Do you live?
1. In an apartment with a garden / terrace
2. In an apartment without an outside space
3. In a house with a garden
4. In a house with no outside space

27. Disposez-vous d'une prise libre dans cette pièce, sans chauffage ou appareil électrique proche, sans fenêtre ou système de climatisation proche, sans meuble proche ou objet dépassant du mur au-dessus ?

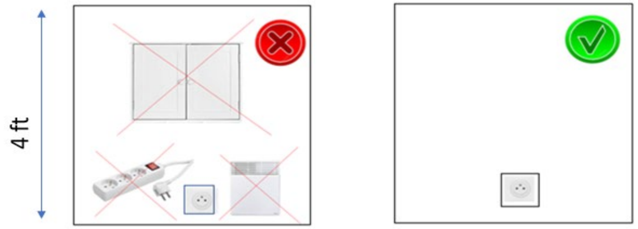

1-Oui
2-No
STOP si code 2

28. Avez-vous l'intention de partir en vacances ou de quitter votre domicile du … au … (durée du test) ?

1. Non, je n'ai pas l'intention de partir.
2. Oui, mais pas plus de 2 jours ou juste un week-end.
3. Oui, au moins 3 ou 4 jours

STOP si code 3

29. Vous êtes ?
1 - Une femme
2 - Un homme

30. Vous vivez ?
1. Dans une zone urbaine
2. A la campagne

31. Vous vivez ?
1. Dans un appartement avec jardin / terrasse
2. Dans un appartement sans espace extérieur
3. Dans une maison avec jardin
4. Dans une maison sans espace extérieur

32. **Which ONE of the following best represents your family situation? (READ LIST, SELECT ONE)**<br>1. Single with no children at home<br>2. Single with children at home<br>3. Married or cohabiting with no children at home<br>4. Married or cohabiting with children at home<br>5. Prefer not to answer	32. **Laquelle des situations suivantes représente le mieux votre situation familiale ? (LISEZ LA LISTE, SÉLECTIONNEZ-EN UNE)**<br>1. Célibataire sans enfant à la maison<br>2. Célibataire avec enfants à la maison<br>3. Marié ou cohabitant sans enfant à la maison<br>4. Marié ou cohabitant avec des enfants à la maison<br>5. Préfère ne pas répondre
33. **Which ONE of the following best describes your employment situation? (SELECT ONE)**<br>1. Full-Time<br>2. Part-Time<br>3. Self Employed<br>4. Student<br>5. Homemaker<br>6. Retired<br>7. Unemployed<br>8. Prefer not to answer	33. **Laquelle des propositions suivantes décrit le mieux votre situation professionnelle ? (CHOISIR UN)**<br>1. Temps plein<br>2. Temps partiel<br>3. Travailleur indépendant<br>4. Étudiant<br>5. Femme / homme au foyer<br>6. Retraité<br>7. Sans emploi<br>8. Préfère ne pas répondre
34. **In general, during the week, excluding evenings, weekends and vacations, how many days do you spend at home?**<br>1.I spend most of my days at home (telecommuting, at home...)<br>2.I spend 1 day away and 4 days at home<br>3.I spend 2 or 3 days at home and 2 or 3 days away<br>4.I spend 1 day at home and 4 days away<br>5.I spend all my days away from home	34. **En général, au cours de la semaine, à l'exclusion des soirées, des week-ends et des vacances, combien de jours passez-vous à la maison ?**<br>1. je passe la plupart de mes journées à la maison (télétravail, à domicile...)<br>2.je passe 1 jour à l'extérieur et 4 jours à la maison<br>Je passe 2 ou 3 jours à la maison et 2 ou 3 jours à l'extérieur.<br>4.Je passe 1 jour à la maison et 4 jours à l'extérieur.<br>5. je passe tous mes jours loin de la maison

D0 questionnaire + Cat's behavior and characteristics (Questionnaire D0 + Caractéristiques et comportement du chat)

1. **What is the breed of your cat?**<br>1. No breed/undetermined/mixed-breed<br>2. Persan<br>3. Bengal<br>4. Norwegian Forest<br>5. Abyssinian<br>6. Bobtail<br>7. British Shortair<br>8. Chartreux<br>9. Maine Coon<br>10. Ragdoll<br>11. Russian Blue<br>12. Scottish Fold<br>13. Siamese<br>14. Sphynx<br>15. Sacred Birman<br>16. Siberian<br>17. European<br>18. Other<br><br>2. **Is your cat?**<br>1. A male not neutered<br>2. A female not spayed<br>3. A male neutered<br>4. A female spayed<br><br>3. **Do you consider your cat to be? (Select all that applies)**<br>1. Aggressive (growling, hissing, scratching, nipping or biting in any circumstances)	1. **Quelle est la race de votre chat ?**<br>1. Pas de race/indéterminée/mixte<br>2. Persan<br>3. Bengale<br>4. Chat norvégien<br>5. Abyssinien<br>6. Bobtail<br>7. British shortair<br>8. Chartreux<br>9. Maine Coon<br>10. Ragdoll<br>11. Bleu russe<br>12. Scottish Fold<br>13. Siamois<br>14. Sphynx<br>15. Sacré de Birmanie<br>16. Sibérien<br>17. Européen<br>18. Autre<br><br>2. **Votre chat est-il ?**<br>1. Un mâle non castré<br>2. Une femelle non stérilisée<br>3. Un mâle castré<br>4. Une femelle stérilisée<br><br>3. **Considérez-vous que votre chat est ? (Sélectionnez tout ce qui s'applique)**<br>1. Agressif (grogne, siffle, griffe, mord ou mordille en toutes circonstances)<br>2. Destructeur

2. Destructive
3. Hyperactive/restless
4. Disobedient
5. Fearfull
6. Excitable
7. Nervous
8. Energetic
9. Noisy/excessive vocalization
10. Depressed?
11. Lively
12. Affectionate
13. Quiet
14. Friendly
15. Active
16. Lazy
17. Demanding attention
18. Playful
19. Relaxed
20. Calm/Chilled
21. Vigilant
22. Calm
23. Sociable
24. Bold
25. Amicable

4. What types of food do you give your cat? (can be selected more than one option)
   1. Dry food
   2. Wet food
   3. Home food
   4. Barf

5. What is his/her actual weight (in Kg) ?
/______//don't know

3. Hyperactif/infatigable
4. Désobéissant
5. Craintif
6. Excitable
7. Nerveux
8. Énergique
9. Bruyant / fait des vocalises excessives
10. Déprimé
11. Vivant
12. Affectueux
13. Discret
14. Amical
15. Actif
16. Paresseux
17. En demande d'attention
18. Joueur
19. Détendu
20. Calme et tranquille
21. Alerte
22. Calme
23. Sociable
24. Audacieux
25. Aimable / conciliant

4. Quels types de nourriture donnez-vous à votre chat ? (vous pouvez choisir plus d'une option)
   1. Aliments secs
   2. Aliments humides
   3. Fait maison
   4. Barf

5. Quel est son poids (en Kg) ?
/______//don't know

6. Please could you click on the figure that reflect more you can body condition

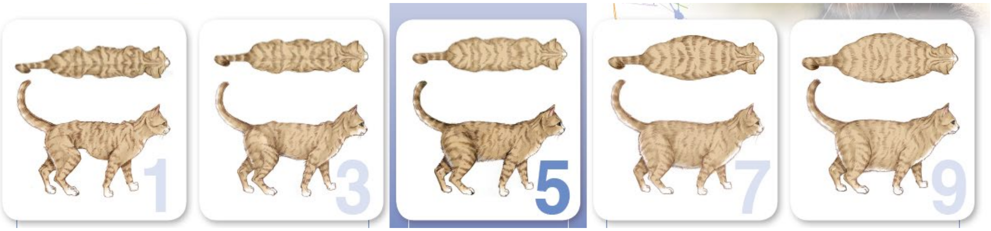

7. Does your cat eat …?
   1. Quickly
   2. Slowly
   3. Neither clowly nor quickly

8. How long does your cat sleep during the day (which is while you are not sleeping)?
   1. Less than 4 hours
   2. Between 4–8 hours
   3. More than 8 hours

9. Is your cat very active at night?
   1. Yes
   2. No

10. Do you provide a litterbox?
   1. Yes
   2. No

11. Does your cat ever eliminate (urine and/or stools) outside the litterbox (if he has access to a litter box) inside the house (exclude situations when the cat has a disease such as diarrhea or urinary tract problems?
   1. Yes, urine
   2. Yes, stools
   3. Yes, urine and stools
   4. No, never

6. Veuillez cliquer sur l’image qui décrit le mieux sa morphologie

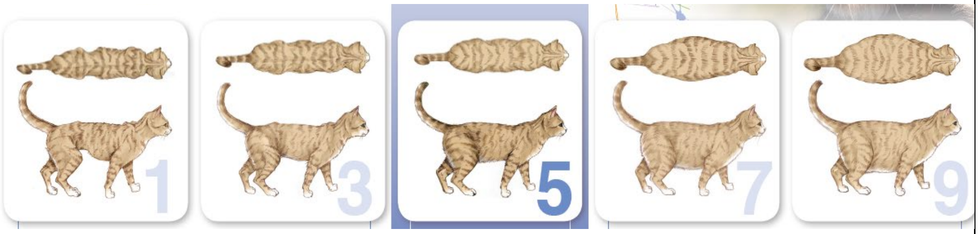

7. Votre chat mange-t-il... ?
   1. Rapidement
   2. Lentement
   3. Ni lent, ni rapidement

8. Combien de temps votre chat dort-il pendant la journée (c'est-à-dire pendant que vous ne dormez pas) ?
   1. Moins de 4 heures
   2. Entre 4 et 8 heures
   3. Plus de 8 heures

9. Votre chat est-il très actif la nuit ?
   1. Oui
   2. Non

10. A-t-il accès à un bac de litière ?
   1. Oui
   2. Non

11. Votre chat fait-il parfois ses besoins (urine et/ou selles) en dehors du bac à litière (s’il y a acces à un bac) à l'intérieur de la maison (à l'exclusion des situations où le chat souffre d'une maladie comme la diarrhée ou des problèmes urinaires) ?
   1. Oui, l'urine
   2. Oui, des selles
   3. Oui, urine et selles
   4. Non Jamais

English	Français
**12.** Is **your cat playful?**<br>1. Yes<br>2. No	12. **Votre chat** est-il **joueur ?**<br>1. Oui<br>2. Non
**13. On average, how much time does your cat plays per day?**<br>1. Doesn´t play<br>2. 5 minutes<br>3. 5 to 15 minutes<br>4. 15 to 30 minutes<br>5. 30 minutes to one hour<br>6. One to two hours<br>7. More than two hours	13. **En moyenne, combien de temps votre chat joue-t-il par jour ?**<br>1. Il ne joue pas<br>2. 5 minutes<br>3. 5 à 15 minutes<br>4. 15 à 30 minutes<br>5. 30 minutes à une heure<br>6. Une à deux heures<br>7. Plus de deux heures
**14. Is your cat keen to explore the home environment?**<br>1. Yes<br>2. No	**14. Votre chat a-t-il envie d'explorer son environnement (la maison)?**<br>1. Oui<br>2. Non
**15. How does your cat behave when visitors come to the house?**<br>1. He/she prefers to stay in a different room<br>2. I put him/her in a different room due to his/her aggressive behavior<br>3. He/she prefers to stay in the same room with visitors but does not interact with them<br>4. He/she prefers to stay in the same room with visitors and interacts with them	15. **Comment votre chat se comporte-t-il lorsque des visiteurs viennent à la maison ?**<br>1. Il/elle préfère rester dans une autre pièce<br>2. Je le/la met dans une autre pièce à cause de son comportement agressif.<br>3. Il/elle préfère rester dans la même pièce que les visiteurs mais n'interagit pas avec eux.<br>4. Il/elle préfère rester dans la même pièce que les visiteurs et interagit avec eux.
**16. Is the behaviour different toward familiar and unfamiliar people?**<br>1. No<br>2. Yes, hides when unfamiliar people arrive and while they are present<br>3. Yes, more aggressive with unfamiliar people<br>4. Yes, more aggressive with owners	16. **Le comportement est-il différent selon qu'il s'agit de personnes familières ou des personnes inconnues ? Plusieurs réponses possibles (modalité « non » excluant autres réponses) ?**<br>1. Non<br>2. Oui, il se cache lorsque des personnes inconnues arrivent et lorsqu'elles sont présentes.

English	Français
5. Yes, avoids unfamiliar people and not the owners e.g. the cat is present nearby but gets away from them if they approach<br>6. Yes, avoids owners and doesn’t avoid unfamiliar people e.g.the cat is present nearby but gets away from them if they approach<br>7. Yes, wants more physical contact with unfamiliar people than with owners<br>8. Yes, wants more physical contact with owners than with unfamiliar people<br>9. Yes, plays more with unfamiliar people<br>10. Yes, plays more with owners<br>11. Yes, explores/investigates (sniffs/smells) more unfamiliar people and/or their items<br>12. Yes, explores/investigates (sniffs/smells) more owners and/or their items	3. Oui, il/elle est plus agressif(ve) avec les personnes inconnues<br>4. Oui, il/elle est plus agressif(ve) plus agressif avec ses propriétaires<br>5. Oui, il évite les personnes inconnues mais pas ses propriétaires (le chat est présent à proximité mais s'éloigne d'eux s'ils s'approchent.)<br>6. Oui, il évite ses propriétaires mais n'évite pas les personnes inconnues(le chat est présent à proximité mais s'éloigne d'eux s'ils s'approchent.)<br>7. Oui, il/elle veut plus de contact physique avec les personnes inconnues qu'avec ses propriétaires.<br>8. Oui, il/elle veut plus de contact physique avec ses propriétaires qu'avec des personnes inconnues.<br>9. Oui, joue davantage avec des personnes inconnues.<br>10. Oui, joue plus avec ses propriétaires<br>11. Oui, explore/renifle davantage les personnes inconnues et/ou leurs objets.<br>12. Oui, explore/ renifle davantage ses propriétaires et/ou leurs objets.
**17. Is your cat quick to approach new people?**<br>1. Yes<br>2. No	**17. Votre chat approche-t-il facilement les inconnus ?**<br>1. Oui<br>2. Non
**18. Has your cat ever bitten anyone?**<br>1. Yes<br>2. No	**18. Votre chat a-t-il déjà mordu quelqu'un ?**<br>1. Oui<br>2. Non
**19. Does your cat groom himself/cleans his haircoat?**<br>1. Yes, in what I think is the normal amount<br>2. Yes, more than he used to do and/or more than I expected a cat to do<br>3. Yes, but less than he used to do and/or less than I expected a cat to do<br>4. Not at all	**19. Votre chat se toilette-t-il ou nettoie-t-il son pelage ?**<br>1. Oui, dans ce que je pense être la fréquence normale<br>2. Oui, plus qu’avant et/ou plus que ce que je pense être normal pour un chat<br>3. Oui, mais moins qu'avant et/ou moins que ce que je pense être normal pour un chat<br>4. Pas du tout
If you selected “2.” Please tell us more:	Si vous avez choisi "2." Veuillez nous en dire plus :

English	Français
20. Does your cat have a skin disease diagnosed by a vet (e.g. allergy, alopecia, dermatitis, fleas, ticks, mites…)?<br>1. Yes<br>2. No	20. Votre chat souffre-t-il d'une maladie de peau diagnostiquée par un vétérinaire (par exemple, allergie, alopécie, dermatite, puces, tiques, acariens...) ?<br>1. Oui<br>2. Non
21. Does your cat have several spots with lack of hair?<br>1. Yes<br>2. No	21. Votre chat présente-t-il plusieurs endroits sans poils ?<br>1. Oui<br>2. Non
22. Does your cat have lack of hair allover his body?<br>1. Yes<br>2. No	22. Votre chat manque-t-il de poils sur tout le corps ?<br>1. Oui<br>2. Non
23. Does your cat have lack of hair in his abdomen and/or inner tights?<br>1. Yes<br>2. No	23. Votre chat manque-t-il de poils au niveau de l'abdomen et/ou de l'intérieur des pattes ?<br>1. Oui<br>2. Non
24. Regarding the size of the scratching area of the cat tree/scratching post/device, is this:<br>1. Smaller than the size of your cat fully streched<br>2. The same as your cat fully streched<br>3. Bigger than the size of your cat fully streched	24. En ce qui concerne la taille de l'arbre à chat/du griffoir/du dispositif de grattage, est-ce que. :<br>1. Plus petit que la taille de votre chat complètement étiré<br>2. La taille que votre chat complètement étiré<br>3. Plus grand que la taille de votre chat complètement étiré
25. Regarding the location of the scratching post it is in:<br>1. The same room as the behaviour problem happens<br>2. A different room from where the behaviour problem happens	25. En ce qui concerne l'emplacement du griffoir dans lequel il se trouve :<br>1. La même pièce que celle où le problème de comportement se produit<br>2. Une pièce différente de celle où le problème de comportement se produit.
26. Does your cat use the cat tree / scratching post / other scratching device that you provide to him?<br>1. No<br>2. Yes	26. Votre chat utilise-t-il l'arbre à chat / le griffoir / le dispositif de grattage que vous lui avez mis à disposition ?<br>1. Non<br>2. Oui

27. Is the the cat tree / scratching post / other scratching device always available for your cat to use?

1. No
2. Yes

28. A.Please could you choose the set of picture that reflect better the seven past day of your cat ?

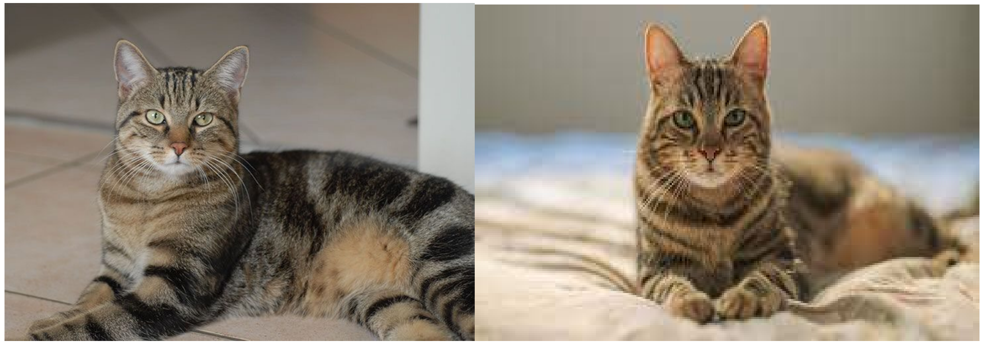

a

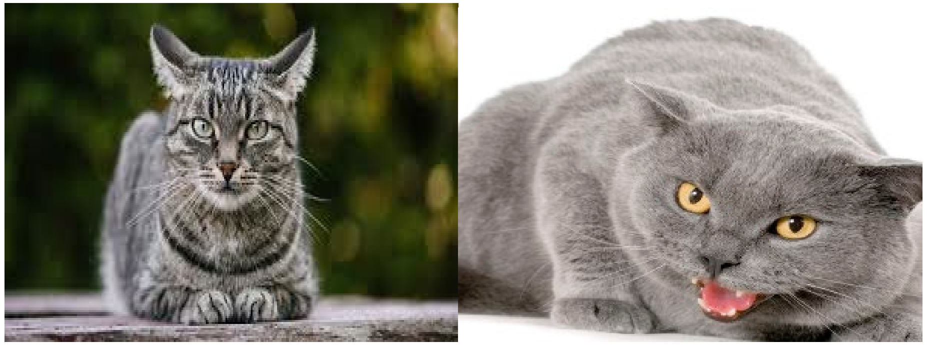

b

27. L'arbre à chat / le griffoir /le dispositif de grattage est-il toujours disponible pour votre chat ?

1. Non
2. Oui

28. A. Pourriez-vous choisir le duo de photos qui reflète le mieux les sept derniers jours de votre chat ?

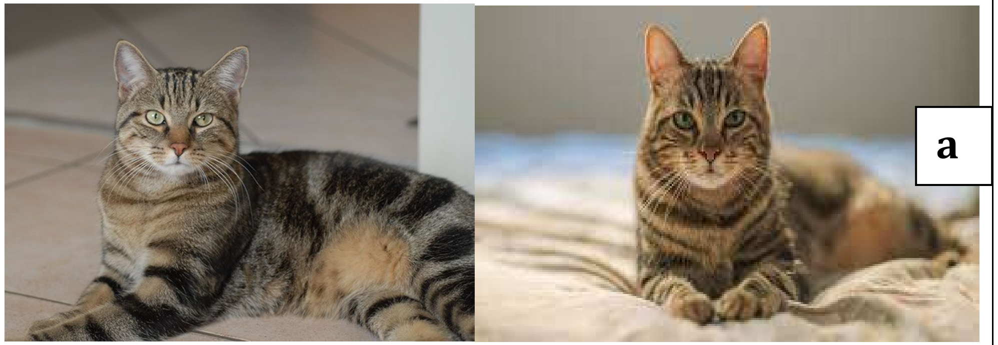

a

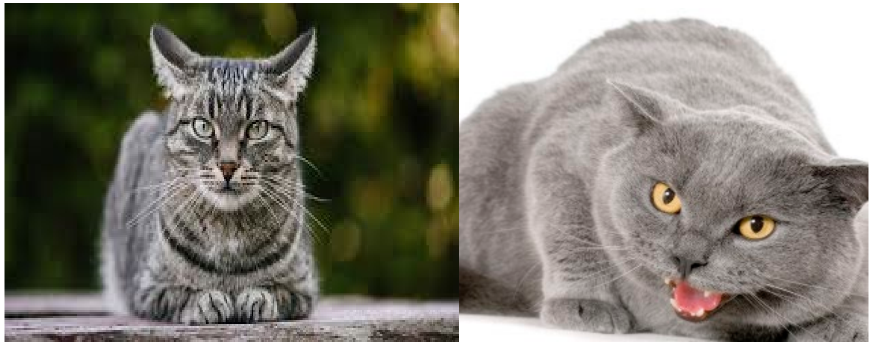

b

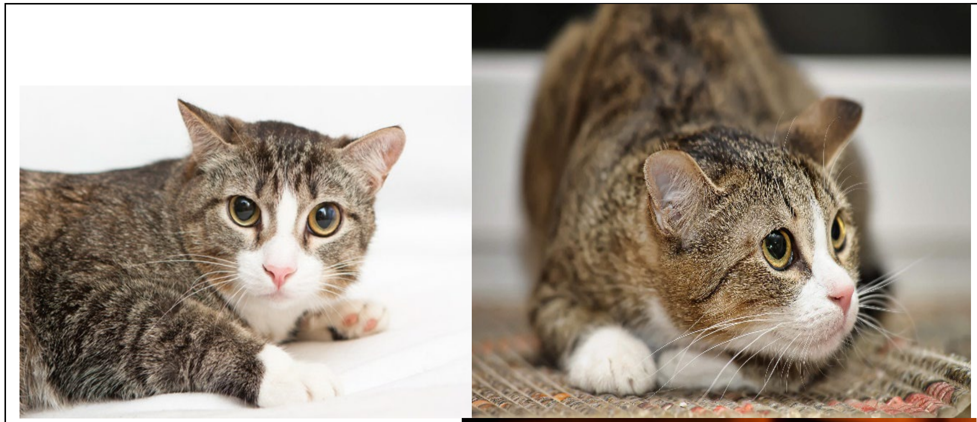

c

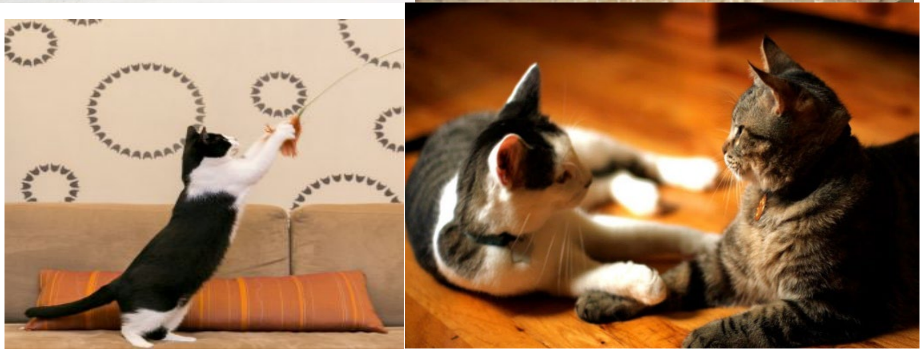

d

29. B. To what extent did you consider this scratching problem is disturbing for you and your household? (from 0 “it is not disturbing at all” to 10 “it is extremely disturbing”)

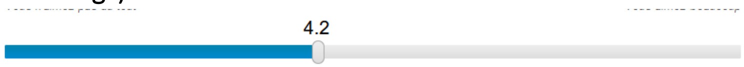

30. C.In the last 7 days, how often have you noticed your cat has made inappropriate scratches (Scratching indoors on vertical surfaces other than the cat tree/scratching post/other scratching device you provide him e.g. sofa, furniture, curtains, door frames...)? The inappropriate scratching could be directly observed or deduced from new damage observed.

6. Every day, more than twice a day

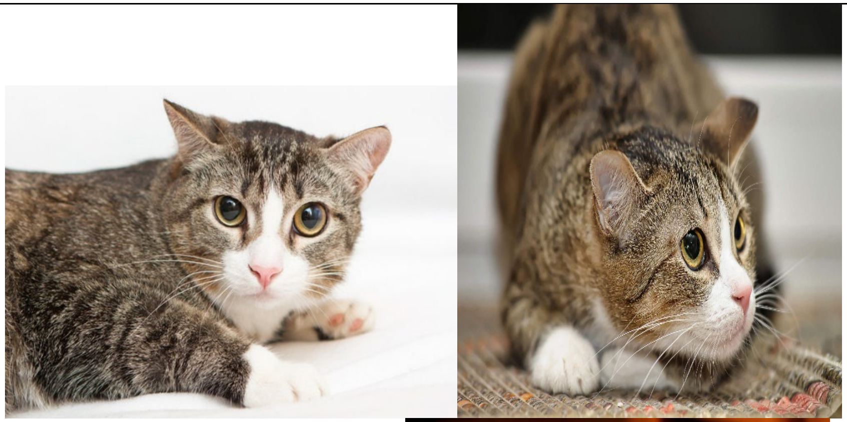

c

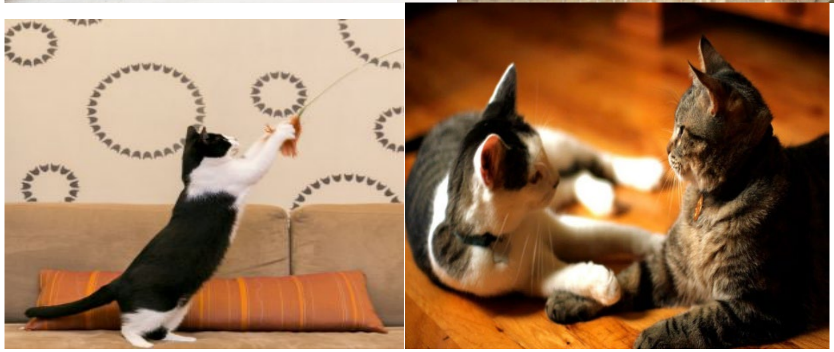

d

29. B. Dans quelle mesure considérez-vous que ce problème de grattage est dérangeant pour vous et votre foyer ? (de 0 "ce n'est pas du tout gênant" à 10 "c'est extrêmement gênant")

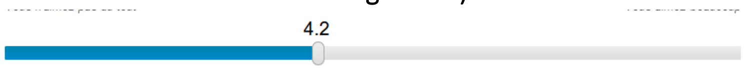

30. C.Au cours des 7 derniers jours, combien de fois avez-vous remarqué que votre chat a fait des griffades inappropriées (grattage à l'intérieur sur des surfaces verticales autres que l'arbre à chat/le griffoir/le dispositif de grattage que vous lui fournissez, par exemple canapé, meubles, rideaux, cadres de porte...) ? Les griffades inappropriés peuvent être observées directement ou déduit de nouveaux dommages observés.

5. Every day, once or twice a day
4. Almost every day
3. Every other day
2. twice a week
1. Once a week
0. Never

31. D_If you have in mind the exact number of times your cat has performed this behavior, please indicate it

/ ___________________/ times per week

32. E_(If different from 0 to frequency) Still regarding [name completed in Q1 BASELINE], disregarding frequency, what do you think is the current average intensity of his scratching? Please place the cursor on this scale to describe the current intensity of this bad habit. the intensity can be evaluate with the duration of the scratch and/or the extend of the damage observed.

Extremely low intensity Extremely high intensity

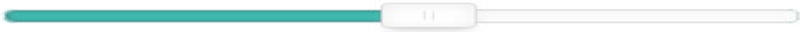

33. Did your cat change the place preferred for the problem scratching?

1. No
2. Yes

Is yes please state from where to where :_________

34. Where does your cat scratches?

1. In many different places (e.g. courtains and sofas and other furniture)
2. Only on courtains
3. Only on sofas
4. Only on chairs

6. Tous les jours, plus de deux fois par jour
5. Tous les jours, une ou deux fois par jour
4. Presque tous les jours
3. Tous les deux jours
2. deux fois par semaine
1. Une fois par semaine
0. Jamais

31. D_Si vous avez en tête le nombre exact de fois où votre chat a eu ce comportement, veuillez l'indiquer.

/ ___________________/ fois par semaine

32. E_(Si différent de 0 à la fréquence) Toujours en ce qui concerne [nom rempli dans Q1 BASELINE], sans tenir compte de la fréquence, quelle est, selon vous, l'intensité moyenne actuelle de ses griffades ? Veuillez placer le curseur sur cette échelle pour décrire l'intensité actuelle de cette mauvaise habitude. *L'*intensité peut être évaluée en fonction de la durée des griffades et/ou de l'étendue des dommages observés.

Intensité extrêmement faible Intensité extrêmement forte

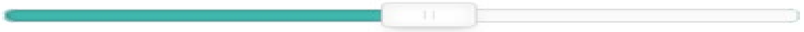

33. Votre chat a-t-il changé l'endroit préféré pour ses griffades problématiques ?

1. Non
2. Oui

Si oui, veuillez préciser de quel endroit à quel endroit :_________

34. Où votre chat griffe-t-il le plus souvent?

1. Dans de nombreux endroits différents (par exemple, sur les rideaux, les canapés et autres meubles)
2. Seulement sur les rideaux
3. Seulement sur les canapés

5. Only on carpets/mats/rugs<br>6. Only on furniture<br>7. Other :	4. Seulement sur les chaises<br>5. Seulement sur les tapis/matelas/moquette<br>6. Uniquement sur les meubles<br>7. Autres :
